# A Biomechanical Analysis of the Interlock Suture and a Modified Kessler-Loop Lock Flexor Tendon Suture

**DOI:** 10.6061/clinics/2017(09)10

**Published:** 2017-09

**Authors:** Wenfeng Yang, Dan Qiao, Yuanfei Ren, Yvjin Dong, Yaohua Shang, Tiehui Zhang

**Affiliations:** Dalian Municipal Central Hospital, Dalian, China

**Keywords:** Tendon Repair, Animal Experimentation, Flexor Tendon

## Abstract

**OBJECTIVE:**

In this work, we attempted to develop a modified single-knot Kessler-loop lock suture technique and compare the biomechanical properties associated with this single-knot suture technique with those associated with the conventional modified Kessler and interlock suture techniques.

**METHODS:**

In this experiment, a total of 18 porcine flexor digitorum profundus tendons were harvested and randomly divided into three groups. The tendons were transected and then repaired using three different techniques, including modified Kessler suture with peritendinous suture, interlock suture with peritendinous suture, and modified Kessler-loop lock suture with peritendinous suture. Times required for suturing were recorded and compared among groups. The groups were also compared with respect to 2-mm gap load, ultimate failure load, and gap at failure.

**RESULTS:**

For tendon repair, compared with the conventional modified Kessler suture technique, the interlock and modified Kessler-loop lock suture techniques resulted in significantly improved biomechanical properties. However, there were no significant differences between the interlock and modified Kessler-loop lock techniques with respect to biomechanical properties, gap at failure, and time required.

**CONCLUSIONS:**

The interlock and modified Kessler-loop lock techniques for flexor tendon sutures produce similar mechanical characteristics *in vitro*.

## INTRODUCTION

Flexor tendon injury is a common clinical trauma. Early postoperative active functional exercise can not only effectively reduce the formation of local adhesions and edema but also facilitate sliding function of the tendon, thereby promoting endogenous tendon healing 1,2. The choice of tendon repair technique plays an important role in tendon healing and postoperative functional exercise. The ideal repair should be easy to perform; provide sufficient strength for healing (over 30 N) 3, which can ensure minimal interference with tendon vascularity; and involve secure suture knots and smooth junction of tendon ends. Currently, various suture techniques 4,5 have been proven to exhibit sufficient resistance to gap formation. However, many techniques are demanding with respect to surgical experience and suturing skills. It is essential to explore a relatively simple tenorrhaphy with good mechanical strength to reduce clinical complications associated with flexor tendon suture.

The modified Kessler suture is regarded as the most widely used clinical suture technique for tendon repair. However, the optimal surgical approach for achieving a successful outcome remains controversial 6. In the interlock stitch suture technique, locking loops produce improved strength and gap resistance after flexor tendon repair compared with the grasping loops used in the modified Kessler suture technique 7. Additionally, various cross-locked cruciate tendon sutures 8 with diverse biomechanical properties have been developed from interlock stitch sutures and have become common alternatives for flexor tendon repair. However, the interlock stitch suture technique is time-consuming and relatively complex, hindering its wide application in clinical practice. Liu et al. 9 modified the Kessler-loop lock suture to enhance resistance strength. However, the use of two knots in the repaired area could lead to a high risk of early failure, suture slippage, and tendon torsion 10.

It is widely accepted that the number of suture passages at the injury site is an extremely important factor in the biomechanical outcomes for different types of tendon sutures. Four-strand suture techniques (such as the Strickland and interlock approaches) have been the most widely investigated techniques in recent decades. In the present study, we modified the Kessler-loop lock suture technique by changing the double-knot suture to a single-knot suture and compared our modified technique with the conventional modified Kessler suture and interlock suture techniques with respect to biomechanical properties and suturing time.

## METHODS

### Tendon Harvest and Repair

Porcine flexor tendons are similar in structure and diameter to human flexor tendons and are thus appropriate for use in biomechanical studies. Eighteen porcine flexor digitorum profundus tendons (circumference: 8-10 mm; approximate weight: 300-400 g) were harvested from the hind legs of adult pigs (Dalian Chuming Co., Ltd.). This study was approved by the Institutional Animal Care and Use Committee of Dalian Medical University. All operations were performed in accordance with international guidelines regarding the care and treatment of experimental animals. The tendons were randomly divided into three groups (with six tendons in each group). In this experiment, 4-0 nylon (Weigao, China) was used for core sutures, and 6-0 nylon (Weigao, China) was used for peritendinous sutures.

**Figure 1:**
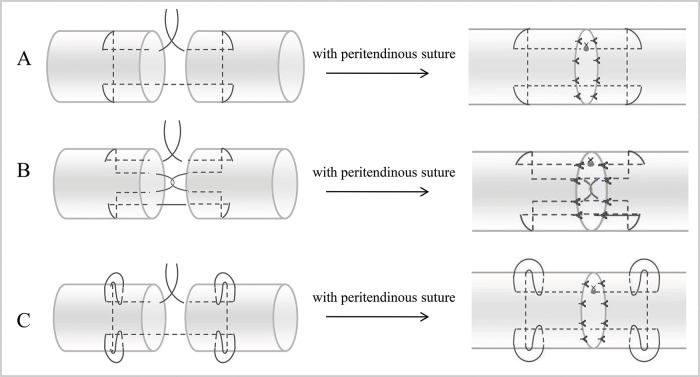
Various suture techniques used for mechanical testing. A. Modified Kessler suture with peritendinous suture (MK); B. Interlock suture with peritendinous suture (IS); C. Modified Kessler-loop lock suture with peritendinous suture (MKL).

The middle of each tendon was transected with a scalpel and then repaired using one of the following techniques: (A) modified Kessler suture with peritendinous suture (MK); (B) interlock suture with peritendinous suture (IS); and (C) modified Kessler-loop lock suture with peritendinous suture (MKL). Details regarding these techniques are presented in Figure 1. During the Kessler suture technique, sutures were tightened to provide proper tension in the first locking loop. A final range of 10-12 mm was maintained for stitches on both sides. Three surgical knots were used for all suture knots, which consisted of six alternating positive and negative single knots. All tendon repairs were performed by a single experienced surgeon within one day. To minimize tissue injury, the surgeon performed the tendon repairs under an operating microscope with a magnification of 10×. Times required for suturing were recorded for each group. After suturing was completed, the repaired tendons were covered with wet gauze (wet with normal saline), stored at 4°C overnight, and subjected to biomechanical testing the following morning.

### Load-to-failure Tests

**Figure 2:**
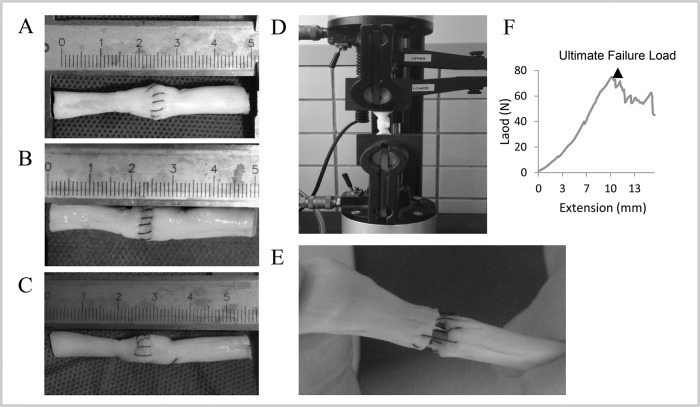
Porcine tendons repaired using various suture techniques. A. Modified Kessler with peritendinous suture (MK); B. Interlock suture with peritendinous suture (IS); C. Modified Kessler-loop lock suture with peritendinous suture (MKL); D. Repaired porcine tendon in mechanical testing; E. Suture failure in mechanical testing; F. Load-deformation curve.

The tendon samples were 50 mm in length with the repair site in the middle. The ends of the tendons were clamped 15 mm from the repair site in a testing machine (Model 5567A, Instron, UK). Bluehill software (Instron, UK) was used to set and record the test parameters (Figure 2). Each tendon was subjected to a preload of 1.0 N and then elongated at a constant velocity of 10 mm/min until the suture site had completely ruptured. The load-to-failure tests were recorded using a camera. During the test, the gap at the repair site was measured using a vernier caliper. In this study, 2-mm gap load was defined as the tensile force needed to produce a 2-mm gap at the repair site. The ultimate load was recorded as the peak force during the stretching process. A computer was used to draw load-displacement curves.

### Statistical Analysis

Stata 12.0 (Computer Sciences Corporation, US) was used for statistical analysis. ANOVA followed by post hoc Tukey tests was used to evaluate differences in2-mm gap load, ultimate failure load, gap at failure, and suture time among the three groups. *p*<0.05 was regarded as indicative of statistical significance.

## RESULTS

### Two-millimeter Gap Load

**Figure 3:**
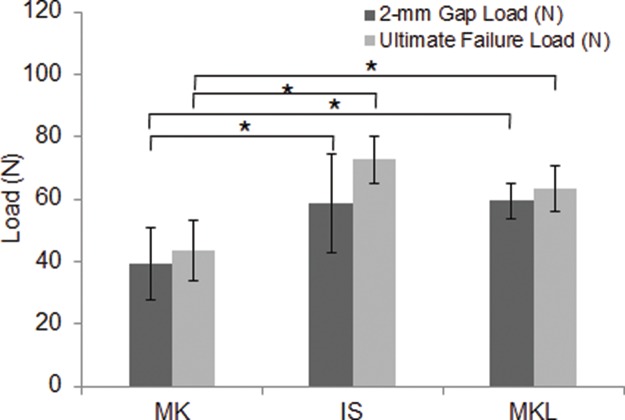
Ultimate failure loads and 2-mm gap loads for different suture techniques. Compared with the MK technique, the IS and MKL techniques resulted in significantly greater 2-mm gap loads and ultimate failure loads. Error bars represent the SD from five porcine flexor tendons. *p<0.05 *vs*. MK.

Two-millimeter gap load is an important index for evaluating resistance strength after tendon repair. During tendon healing, the formation of a gap of 2 mm or more increases the risks of tendon adhesion and impacts tendon healing. As shown in Tables 1 and 3 and Figure 3, compared with the MK technique, both the IS technique (*p*=0.011) and the MKL technique (*p*=0.028) could produce significantly improved 2-mm gap loads. The IS and MKL techniques did not significantly differ with respect to 2-mm gap load (*p*=0.851).

### Ultimate Failure Load

Ultimate failure load is another essential indicator for describing the biomechanical properties of tendon repair. As shown in Tables 1 and 3 and Figure 3, compared with the MK technique, both the IS technique (*p*<0.001) and the MKL technique (*p*=0.006) could produce significantly improved ultimate failure loads. However, the IS and MKL techniques did not significantly differ with respect to ultimate failure load (*p*=0.220).

### Failure Profile

As shown in Table 4, suture breakage always occurred in the IS group. The breakage locations were usually at the gap site in the peritendinous suture, although a small portion of ruptures occurred at the root of a knot. Compared with the MK and MKL groups, the IS group had less suture pullout and knot failure but a higher risk of suture breakage.

### Gap at Failure

**Figure 4:**
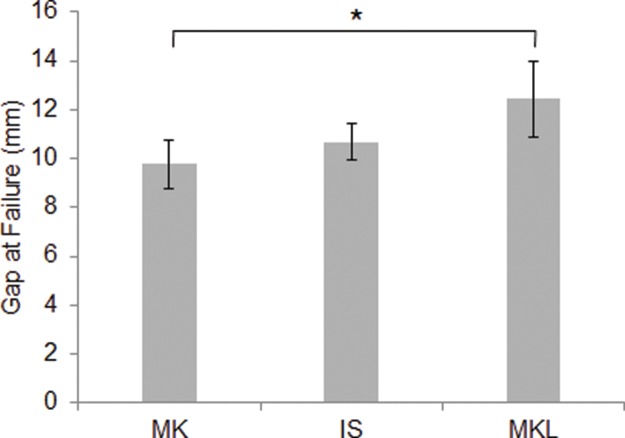
Gaps at failure for different suture techniques. The largest gap at failure was seen in the MKL group (12.4±1.5 mm), whereas gaps at failure were similar for the MK and IS techniques (9.8±1.0 mm *vs*. 10.7±0.7 mm, respectively). *p<0.05 *vs*. MK.

As shown in Tables 2 and 3 and Figure 4, the largest gap at failure was observed in the MKL group, whereas statistically similar findings were obtained for the MK and IS groups (*p*=0.428).

### Time Required for the 3Suture Techniques

**Figure 5:**
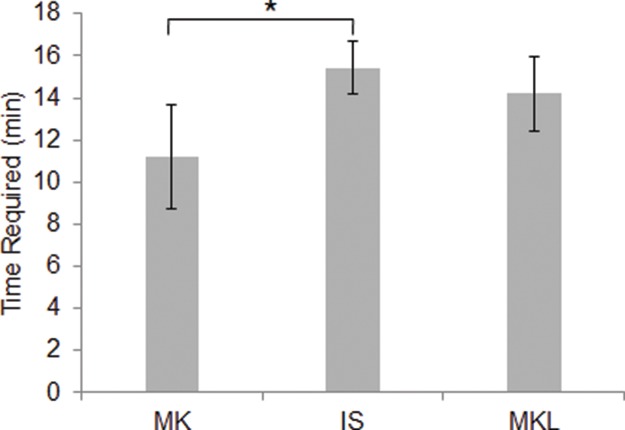
Times required for different suture techniques. Less time was required for the MK technique (11.2±2.5 min) than for the IS technique. There was no significant difference between the times required for the IS and MKL techniques. *p<0.05 *vs*. MK.

As shown in Tables 2 and 3 and Figure 5, the MK technique was faster to perform than the IS technique (*p*=0.011). There was no significant difference in suturing time between the IS and MKL groups (*p*=0.577).

## DISCUSSION

The early mobilization of reconstructed flexor tendons is highly significant for tendon recovery. By establishing stress orientation, early active movement can promote the regular arrangement of collagen fibers and break adhesion between the impaired tendon and surrounding tissues. Various suture techniques, such as the MK, double-strand Kessler suture, Silfverskiold 11, Strickland 12, cross-locked cruciate suture 13, Tsuge 14, and combined Kessler-Tsuge 15 techniques, have been used in clinical practice. However, research has demonstrated that these approaches produce different outcomes. Early active mobilization after tendon repair imposes high load at the repair site and requires resistance strength against a 2-mm gap load of more than 30 N 16. Certain of the most commonly used suture methods 17 do not allow the repaired tendon to withstand over 30 N of tension. Although other repair techniques 18 can provide adequate strength, these techniques require complex surgical procedures or affect blood supply and tendon healing.

Suture methods for flexor tendons mainly involve grasping loops and locking loops. Compared with grasping loops, locking loops improve the strength and gap resistance of the tendon repair by tightly holding tendon bands together. It has also been reported that a grasping loop-locking loop combination can maximize the advantages of both suture techniques and dramatically improve the biomechanical properties achieved in tendon repair 19. In the current work, both the IS and MKL suture techniques exhibited the advantages of locking and grasping loops and produced significantly improved 2-mm gap loads and ultimate tensile strengths relative to the MK technique. The IS and MKL approaches did not significantly differ with respect to 2-mm gap load, ultimate failure load, gap at failure, or time required for suturing.

However, different biomechanical properties are observed for the IS and MKL techniques. To withstand greater tensile force with an intra-tendinous locking structure and stitches running through the tendon fibers, the IS technique is based on the grasping loops used in the Kessler suture technique combined with locking loops in the tendon’s core. However, the crossing shear force in the locking suture of the IS pattern increases the risk of suture breakage. If preload is insufficient during tendon suturing, the rotational stress caused by loose sutures typically leads to twisting and instability of the injured tendon ends; this phenomenon impacts endogenous tendon healing. Therefore, the IS technique is more suitable for experienced surgeons and requires the use of high-quality sutures to withstand tensile forces.

In the Kessler-loop lock suture technique, the tendon is locked by friction between loop sutures and the tendon, with the objective of withstanding greater tensile force and preventing gap formation 20. This approach requires fewer knots than the MK technique. The cross-linked collagen fibers in the tendon generate upward force on the suture, reducing tendon slippage and splitting. The low tension between the injured ends of the tendon may be associated with better tendon healing, although further research is necessary to confirm this possibility.

Therefore, we altered the double-strand Kessler-loop lock suture approach by utilizing single-strand sutures and performed suturing under an operating microscope with a magnification of 10×. This approach is easier for less experienced surgeons to understand and accept. Since this approach does not require special sutures, it is relatively readily applied in clinical practice.

Nevertheless, there are certain limitations of our study. This investigation involved the use of devitalized porcine flexor tendons that may have different biomechanical properties than living tissues. However, in the future, our results could be evaluated in animal models and via clinical studies. Our approach was compared with only the Kessler and interlock suture techniques; this comparison could be extended to other representative 4-strand suture techniques, such as the double-strand Kessler and Strickland approaches.

## AUTHOR CONTRIBUTIONS

Yang W performed biomechanical testing, analyzed data and wrote the manuscript. Qiao D and Ren Y harvested porcine flexor digitorum profundus tendons. Dong Y and Shang Y contributed to writing the manuscript. Zhang T directed the work. All authors read and approved the final version of the manuscript.

## Figures and Tables

**Table 1:** The 2-mm gap loads and ultimate failure loads for three suture techniques.

Group	2-mm Gap Load (N)		Ultimate Failure Load (N)	
	Mean	SD	Mean	SD
MK	39.1	11.6	43.4	9.6
IS	58.8	15.9	72.6	7.7
MKL	59.4	5.8	63.4	7.2

**Table 2:** The gaps at failure and times required for three suture techniques.

Group	Gap at Failure (mm)		Time Required (min)	
	Mean	SD	Mean	SD
MK	9.8	1.0	11.2	2.5
IS	10.7	0.7	15.4	1.3
MKL	12.4	1.5	14.2	1.8

**Table 3:** *p*-values for comparisons between flexor tendon suture techniques.

Comparison	2-mm Gap Load	Ultimate Failure Load	Gap at Failure	Time Required
MK vs. IS	0.011	<0.001	0.428	0.011
MK vs. MKL	0.028	0.006	0.008	0.070
IS vs. MKL	0.851	0.220	0.075	0.577

**Table 4:** Failure modes for three suture techniques.

Group	Suture Breakage	Suture Pullout	Knot Failure
MK (n=6)	n=4	n=4	n=3
IS (n=6)	n=6	n=2	n=1
MKL (n=6)	n=4	n=4	n=2
